# Supplementary material for: Investigating Both Mucosal Immunity and Microbiota in Response to Gut Enteritis in Yellowtail Kingfish
Source: Microorganisms. 2020 Aug 20;8(9):1267. doi: 10.3390/microorganisms8091267 (PMC7565911; doi:10.3390/microorganisms8091267)

Supplementary figures

Figure S1: Rarefaction plot of all samples used in this study showing sufficient sequencing depth at 16,255 reads/sample.


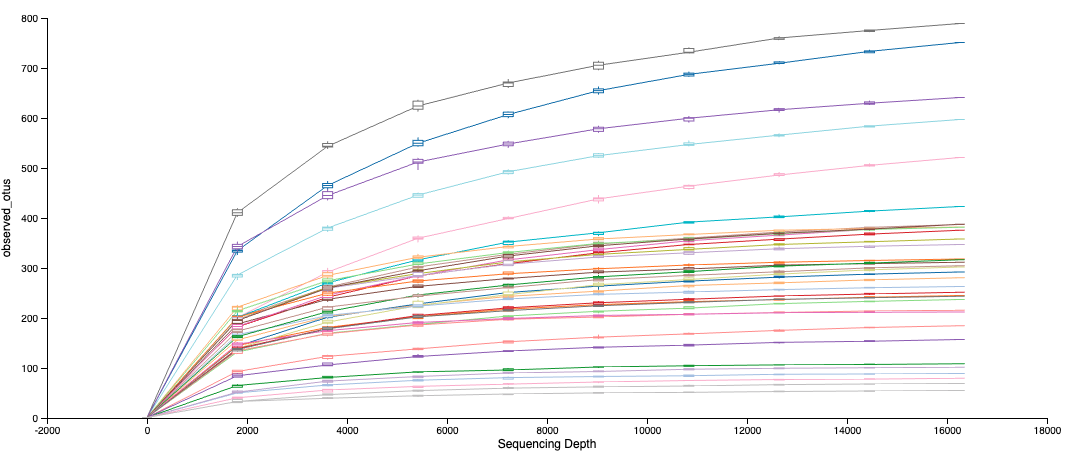


Figure S2: PCoA plot representing the gene expression of both gut and skin samples of all fish used in this study.


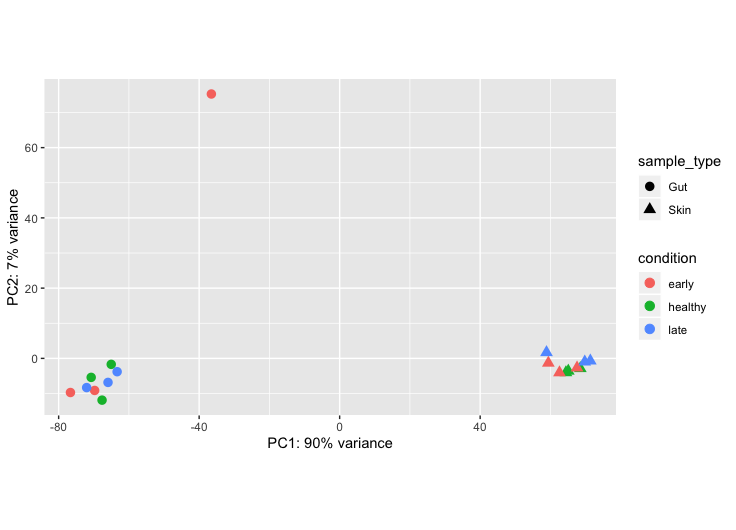


Figure S3: Gut global bacterial community changes associated with gut enteritis. PCoA plot based on the unweighted Unifrac distance matrix.

Figure S4: Skin global bacterial community changes associated with gut enteritis. PCoA plot based on the unweighted Unifrac distance matrix.

Figure S5: Boxplot representing the Pielou’s evenness in the gut microbiota for the different health status. Statistical differences were assessed using a Kruskall-Wallis test.

Figure S6: Boxplot representing the Chao1 richness in the gut microbiota for the different health status. Statistical differences were assessed using a Kruskall-Wallis test.

Figure S7: Boxplot representing the Pielou’s evenness in the skin microbiota for the different health status. Statistical differences were assessed using a Kruskall-Wallis test.


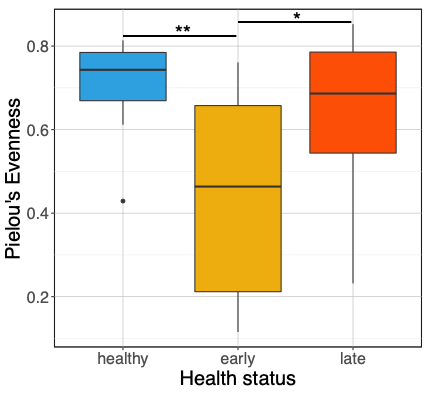


Figure S8: Boxplot representing the Chao1 richness in the skin microbiota for the different health status. Statistical differences were assessed using a Kruskall-Wallis test.


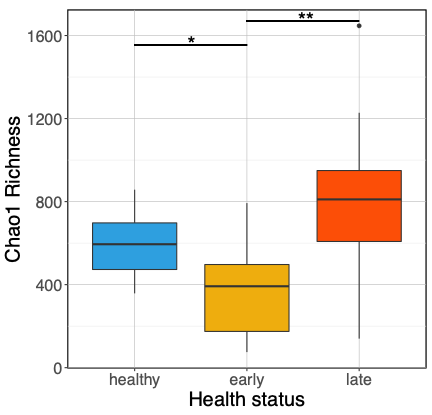


Figure S9: Representation of genes involved in the ECM-receptor interaction pathway in the fish at the late stage of the disease. Genes in red were upregulated, in blue were downregulated and in green were not statistically differentially expressed.


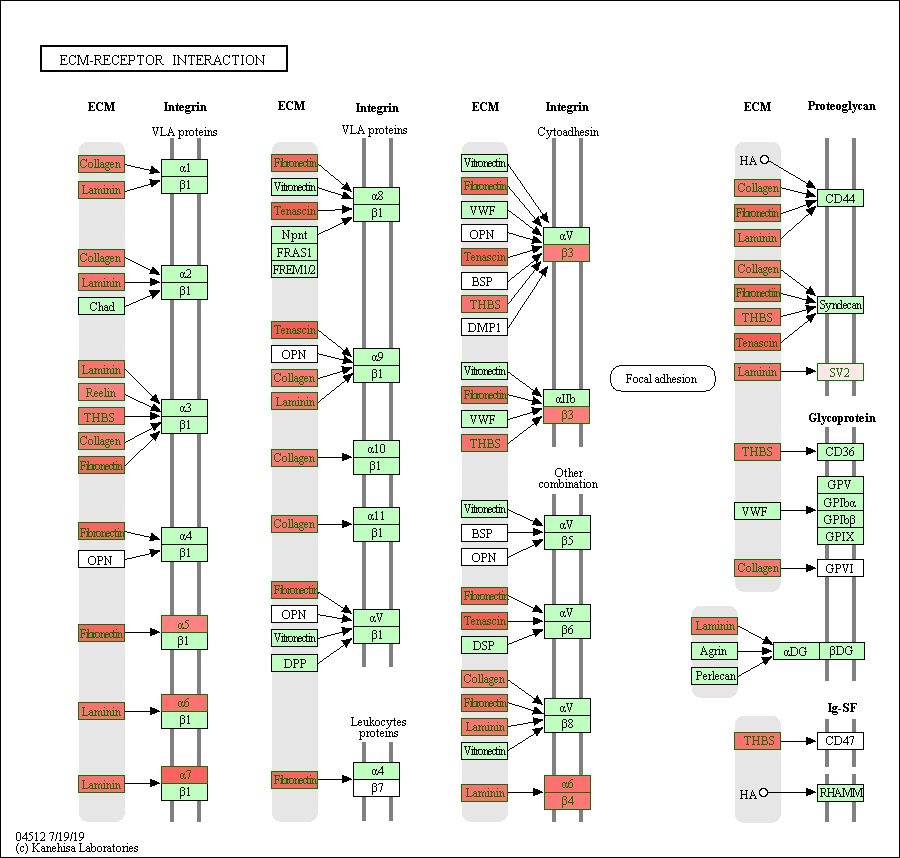

Supplement: Supplementary file 1 [file microorganisms-08-01267-s001.zip › Supplementary figures.docx]
